# Stability of Zinc Finger Nuclease Protein Is Enhanced by the Proteasome Inhibitor MG132

**DOI:** 10.1371/journal.pone.0054282

**Published:** 2013-01-24

**Authors:** Suresh Ramakrishna, Young-Hoon Kim, Hyongbum Kim

**Affiliations:** Graduate School of Biomedical Science and Engineering/College of Medicine, Hanyang University, Seoul, South Korea; Center for Genomic Regulation, Spain

## Abstract

**Background:**

Zinc finger nucleases (ZFNs) are powerful tools for gene therapy and genetic engineering. The characterization of ZFN protein stability and the development of simple methods to improve ZFN function would facilitate the application of this promising technology. However, the factors that affect ZFN protein stability and function are not yet clear. Here, we determined the stability and half-life of two ZFN proteins and examined the effect of MG132 (carbobenzoxyl-leucinyl-leucinyl-leucinal-Hl), a proteasome inhibitor, on ZFN-mediated gene modifications.

**Methodology/Principal Findings:**

ZFN proteins were expressed in 293T cells after transfection of ZFN-encoding plasmids. We studied two ZFN pairs: Z-224, which targets the *CCR5* gene, and K-230, which targets a region 230 kbp upstream of *CCR5*. Western blotting after treatment with cycloheximide showed that the half-life of these ZFN proteins was around two hours. An immunoprecipitation assay revealed that the ZFN interacts with ubiquitin molecules and undergoes polyubiquitination *in vivo*. Western blotting showed that the addition of MG132, a proteasomal inhibitor, increased ZFN protein levels. Finally, a surrogate reporter assay and a T7E1 assay revealed that MG132 treatment enhanced ZFN-directed gene editing.

**Conclusions:**

To our knowledge, this is the first study to investigate ZFN protein stability and to show that a small molecule can increase ZFN activity. Our protein stability study should lay the foundation for further improvement of ZFN technology; as a first step, the use of the small molecule MG132 can enhance the efficiency of ZFN-mediated gene editing.

## Introduction

Zinc finger nucleases (ZFNs) are artificial restriction enzymes that are comprised of custom-designed zinc finger proteins and a nuclease domain derived from the FokI endonuclease [1]. Zinc finger proteins can be designed to bind to specific sequences of DNA, allowing ZFNs to induce double- or single-strand breaks [2], [3] in specific regions of a genome [1], [4]. Such ZFN-induced breaks can induce mutations in genes of interest through error-prone non-homologous end joining [1], [5]–[7] or lead to the modification of genes by homologous recombination in the presence of donor DNA [8]–[10] or single-stranded oligonucleotides [11]. Such targeted-genome editing approaches have been carried out across a variety of species, including fruit flies [5], [12], nematodes, fish [6], rats [13], plants [14], [15], and human cells [7], [16]. Genetic modifications derived from ZFN technology greatly facilitate the investigation of biological processes. In addition, ZFN technology is actively being studied as a means of advanced gene therapy to correct pathogenic genes [17]–[22].

One of the biggest roadblocks to the application of ZFNs is the relatively low efficiency of gene editing by ZFNs. Thus, several approaches have been undertaken to improve ZFN function [23]–[26]. For example, the ZFN nuclease domain has been modified to improve ZFN activity and specificity [24], [26]. Additionally, modifying the culture temperature caused a significant increase in ZFN activity [23]. Furthermore, our group recently reported a simple method to enrich cells that contain ZFN-induced gene disruptions [25]. Given that these simple methods to improve the ZFN function have facilitated the use of ZFNs, the identification of small molecules that increase ZFN function should likewise efficiently facilitate the application of ZFNs. However, such small molecules have yet to be identified.

It has been observed that ZFN protein levels are directly correlated with ZFN function [23], [25]. Culturing the cells at low temperature increases ZFN function at least in part because ZFN protein levels increase [23]. We also observed that cell populations that are enriched with gene-disrupted cells have high ZFN levels as compared to control cells [25]. Recently, direct delivery of ZFN proteins has been shown to be safer associated with negligible off-target effects [27]. These ZFN proteins could penetrate the cells without any additional cell-penetrating peptide sequences and were able to transduce into several cell types including those that are hard to transfect. However, due to degradation of the delivered protein, it was necessary to treat the cells several times with the ZFN protein to obtain significant genetic modifications. Thus, we postulated that stabilizing the ZFN protein could enhance ZFN function. However, ZFN stability and the factors that affect it have yet to be investigated.

Proteins are in a continual state of flux between synthesis and degradation in a cell [28], [29]. The ubiquitin proteasome pathway (UPP) is one of the major cellular regulatory mechanisms involved in protein turnover and half-life [28], [30]–[32]. UPP plays a key role in eliminating intracellular proteins in eukaryotes, especially misfolded cellular proteins [28], [33]. During ubiquitination, a post-translational modification that targets proteins for degradation by the 26S proteasome, multiple ubiquitin molecules are covalently attached to targeted proteins. This process is catalyzed by a three step cascade mechanism, which involves a ubiquitin activating enzyme (E1), a ubiquitin conjugating enzyme (E2), and a ubiquitin ligase (E3) [28], [33]. E1 activates ubiquitin molecules by the formation of an ATP-dependent thiol ester bond between the C-terminus of ubiquitin and the active cysteine site of the E1 enzyme. Activated ubiquitin is transferred to the active cysteine site of the E2 enzyme. Ultimately, E3 catalyzes the transfer of ubiquitin molecules to a lysine residue, ultimately forming polyubiquitin chains on the protein that is destined for degradation. Finally, ubiquitinated proteins are directed into the 20S core proteolytic chamber in an ATP-dependent manner for 26S proteasomal degradation [28], [31], [33]. Small chemical molecules, such as synthetic, cell-permeable peptide aldehydes that form covalent adducts with the 20S proteasome and inhibit its peptidase activities, have been developed [29], [30]. Synthetic proteasome inhibitors are peptide aldehydes which are broadly used as inhibitors for both Serine and Cysteine proteases. Several proteasome inhibitors that can enter the cells and block protein degradation pathway have been identified. Among them, the proteasome inhibitor MG132 is the most widely used commercial inhibitor for regulating the UPP [29]. Because ZFN levels are directly proportional to ZFN activity, we wished to check ZFN proteolysis with MG132 and determine the effects on ZFN-mediated gene disruption.

Here, for the first time, we investigated ZFN protein stability. We found that ZFNs undergo proteasomal degradation and that MG132 increases ZFN levels, leading to enhanced genetic modifications by the ZFNs. Our protein stability study should lay the foundation for the advancement of ZFN technology; furthermore, the identification of MG132 as a small molecule that increases ZFN function is expected to facilitate the use of ZFNs.

## Results

### The proteasome inhibitor MG132 increases ZFN protein levels

**Figure 1:**
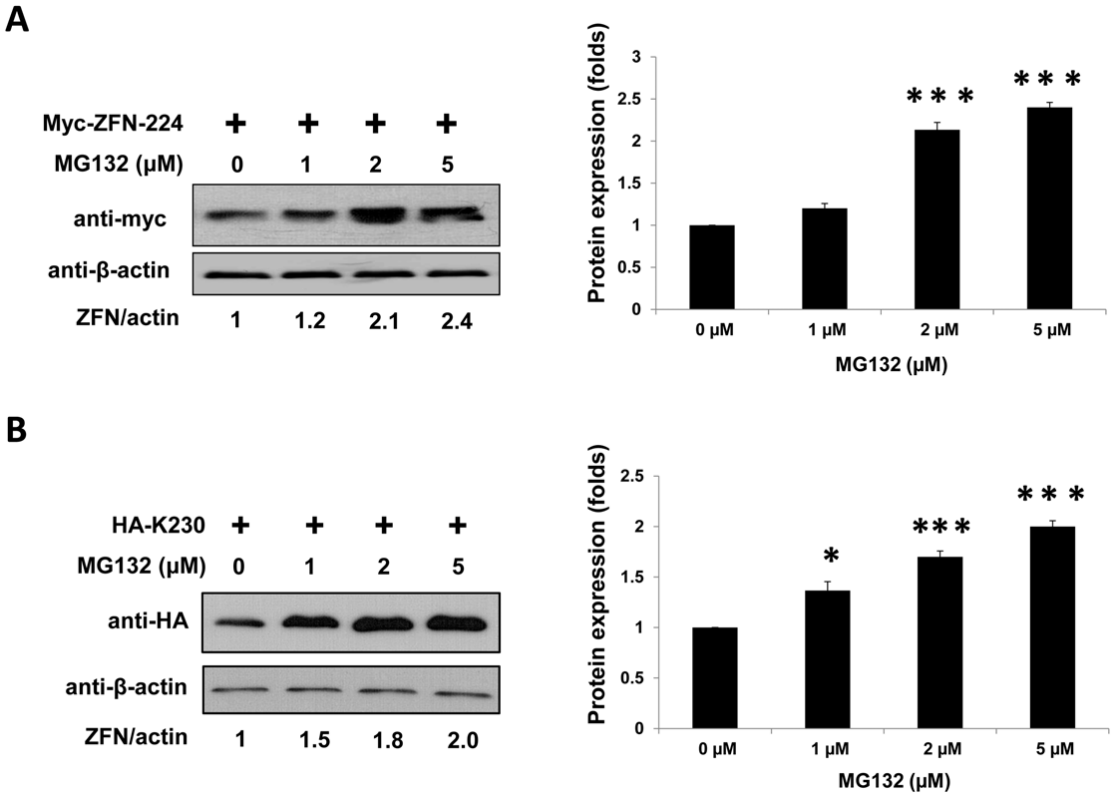
Proteasome inhibitor MG132 increases ZFN protein levels. 293T cells were transfected with equal concentrations of ZFN-encoding plasmids (**A**, myc-ZFN-224; **B**, HA-K230) and treated with various concentrations of MG132 for 16 hours. The ZFN levels were determined by Western blot. As an internal control, β-actin was used. n = 3. *P<0.05, ***P<0.001.

To investigate whether ZFNs are regulated by the UPP degradative pathway, we examined ZFN protein levels in the presence or absence of the proteasomal inhibitor MG132. For these experiments, we studied two pairs of ZFNs: ZFN-224, which targets the *CCR5* gene, and K-230, which targets a region 230 kbp upstream of *CCR5* [3], [34], [35]. We transfected 293T cells with equal amounts of ZFN-224- or K230-encoding constructs, and then treated the cells with several concentrations of MG132 for 16 hrs, after which the cells were harvested. We found that MG132 increased ZFN levels in a dose-dependent manner (**Figure 1A, B**). ZFN protein levels increased 2.4 (ZFN-224)- or 2.0 (K230)-fold as a result of treatment with 5 µM MG132 as compared to levels in MG132-untreated cells. (In this study, the MG132 concentration was restricted to 5 µM because we observed decreased cell viability at higher concentrations.) The increase in ZFN levels as a result of treatment with a proteasome inhibitor led us to speculate that the UPP was involved in regulating ZFN protein turnover.

### ZFN undergoes proteolysis through the UPP

**Figure 2:**
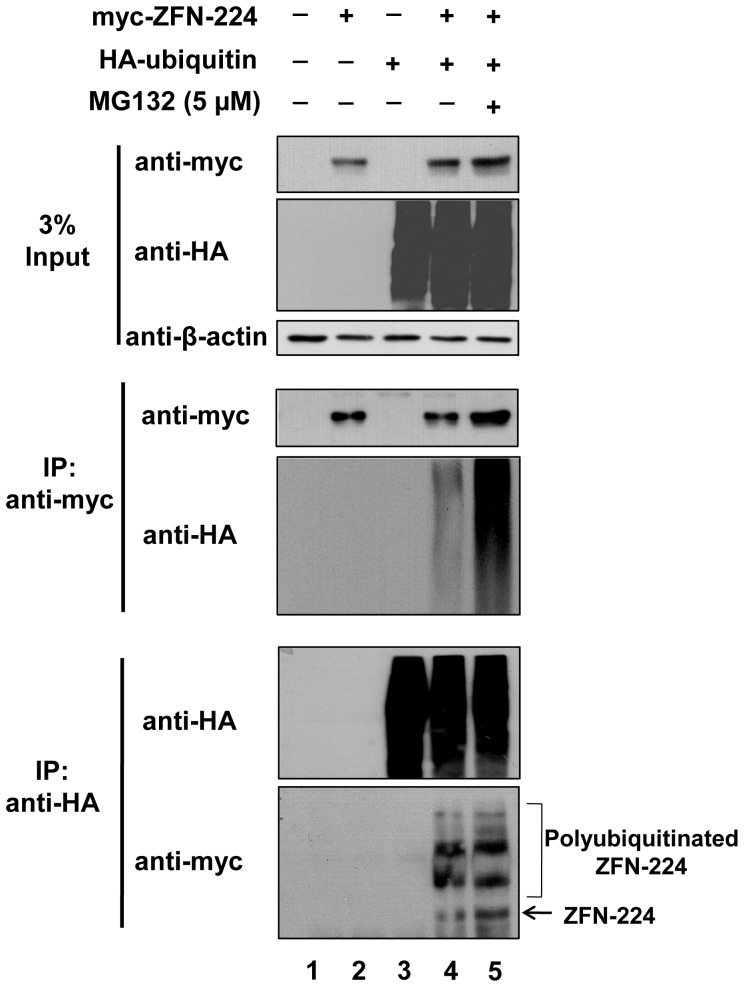
*In vivo* ubiquitination of a ZFN. 293T cells were transfected with pcDNA3-myc-ZFN-224 and pEFIRES-HA-ubiquitin separately and together. ZFN ubiquitination was confirmed by co-immunoprecipitation with an anti-myc antibody and immunoblotting with an anti-HA antibody. To cross confirm, co-immunoprecipitation was performed using anti-HA antibody and immunoblotting with an anti-myc antibody. MG132 treatment further increased ZFN ubiquitination, as shown by the increased amounts of the high molecular weight smear, which represents polyubiquitin coupled with the ZFN.

To investigate whether ZFN protein turnover is regulated by the UPP, we determined whether ubiquitin interacts with ZFN-224 *in vivo*. We co-transfected 293T cells with HA-tagged ubiquitin and myc-tagged ZFN-224 and then performed an immunoprecipitation assay using an anti-myc antibody followed by immunoblotting using an anti-HA antibody. Our data showed a characteristic high molecular weight smear of polyubiquitin molecules conjugated to ZFN-224 in these co-transfected cells (**Figure 2** **lane 4, upper panel**). In contrast, we did not detect ubiquitin molecules in cells transfected with ZFN-224 alone (**Figure 2** **lane 2**). We also carried out a reciprocal immunoprecipitation assay using an anti-HA antibody followed by immunoblotting with an anti-myc antibody. The results again showed that the ZFN-224 was associated with polyubiquitin chains (**Figure 2** **lane 4, lower panel**).

We next examined the effect of MG132 on ZFN ubiquitination. The addition of MG132 (5 µM) to the co-transfected cell culture for 16 hrs resulted in increased accumulation of ubiquitinated ZFN-224 in 293T cells (**Figure 2** **lane 5**). This result suggests that the ZFN-224 protein interacts with ubiquitin molecules and undergoes polyubiquitination through the UPP.

### ZFN half-life

**Figure 3:**
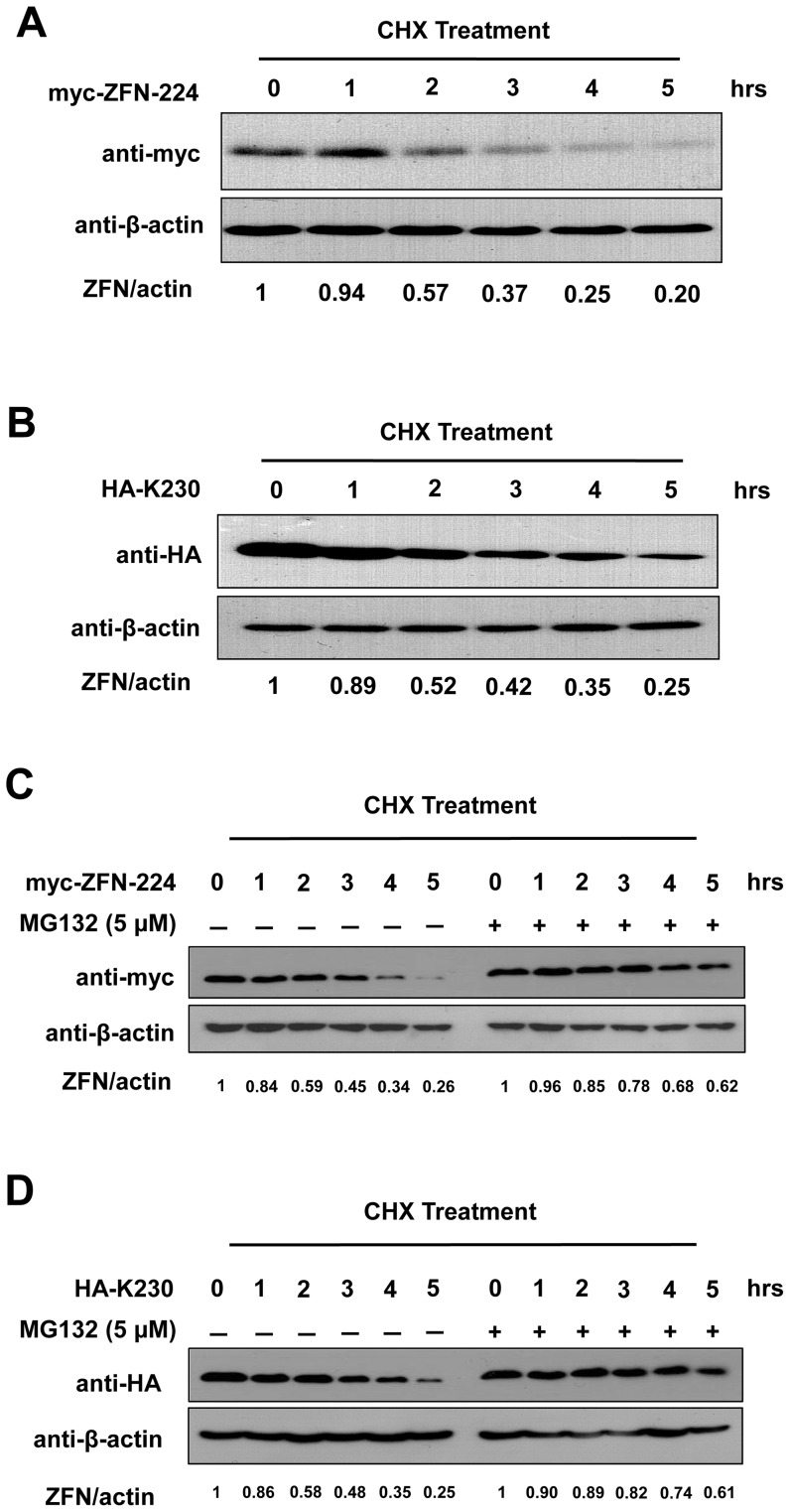
Proteasome inhibitor MG132 extends the ZFN half-life. After transfection of ZFN-encoding plasmids (**A** and **C**, myc-ZFN-224; **B** and **D**, HA-K230) into 293T cells and treatment with CHX (200 µg/mL) for different time intervals, the ZFN levels in these cells were analyzed. The duration of CHX treatment is indicated above the blot. Experiments were performed either in the absence or presence of MG132 (5 µM) as indicated on the figure. Each experiment was repeated at least three times.

We next investigated the relative half-life of the ZFN proteins in transfected cells. We transfected 293T cells with myc-tagged ZFN-224 or HA-tagged K230 and treated them with cycloheximide (CHX), an inhibitor of new protein synthesis. At 48 hours post transfection, cells were treated with CHX (200 µg/mL) for the indicated amount of time and harvested periodically. Western blotting showed that ZFN protein levels were reduced significantly within 5 hrs of CHX treatment (**Figure 3A, B**). The initial ZFN protein level was reduced to half within 2 hours of treatment with CHX. Thus, ZFNs exhibited a relatively short half-life about 2 hours in such transfected cells.

### ZFN half-life is extended by proteasome inhibitor MG132

To determine whether proteasome activity influences the ZFN protein's lifespan, we investigated the effect of MG132 on the ZFN half-life. 293T cells were transfected with an equal amount of plasmids encoding ZFN-224 or K230 and incubated with or without MG132 (5 µM) for 16 hours prior to incubation with CHX (200 µg/mL). The cells treated with CHX were harvested at regular time intervals and examined by Western blot analysis to determine the expression level of the ZFN-224 or K230 protein. MG132 treatment extended the relative half-life of ZFNs in transfected cells (**Figure 3C, D**). In the presence of MG132, the initial level of ZFN protein was not reduced to half even at 5 hours; in contrast, in MG132 untreated cells, ZFN levels are reduced by three quarters at this time point. As a control, the expression of β-actin was checked to confirm equal loading of samples. Thus, MG132 treatment stabilizes ZFN levels by extending the protein's half-life.

### Proteasome inhibitor MG132 enhances ZFN activity

**Figure 4:**
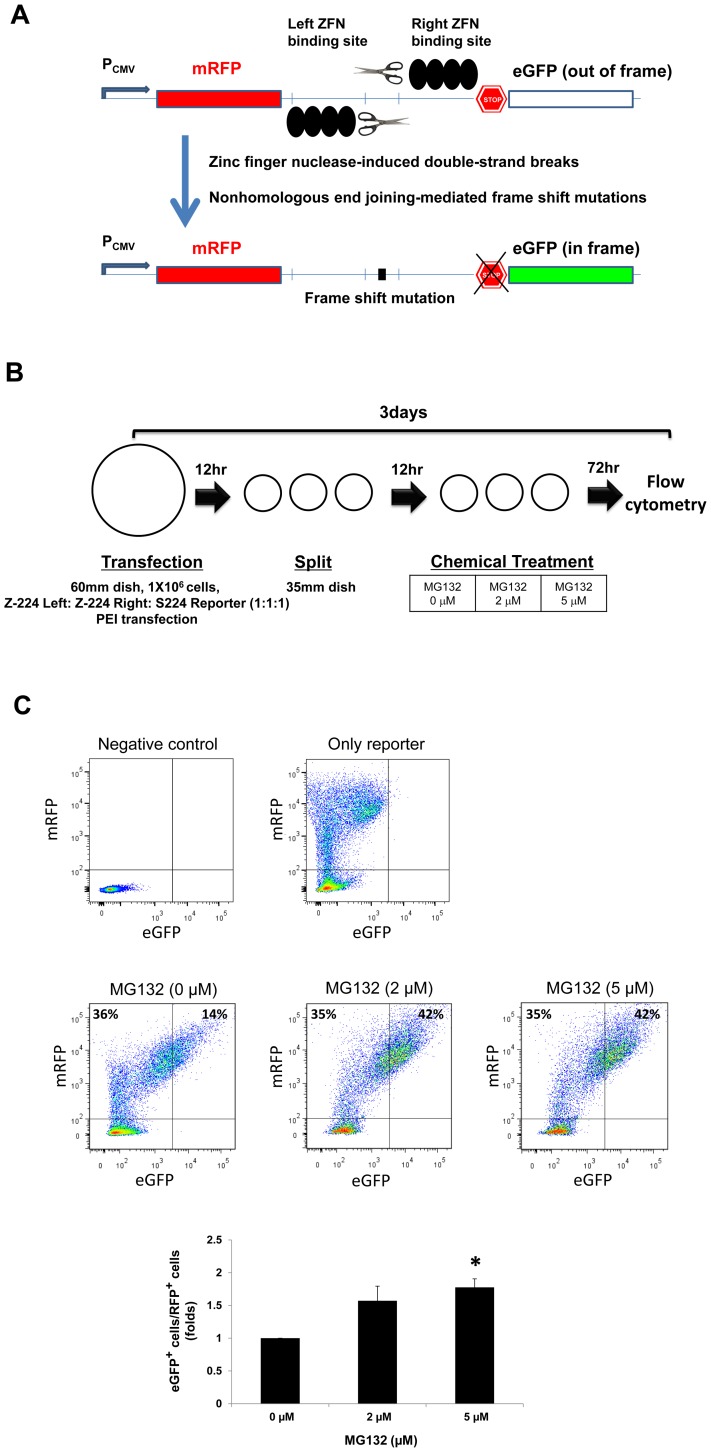
MG132 enhances ZFN function. (**A**) Schematic of the surrogate reporters used for quantifying the effect of MG132 on ZFN function. The reporter was constructed by introducing the mRFP gene (in-frame), a ZFN target sequence, and eGFP gene (out-of-frame) into pRGS vector. Frame shift mutations created by non-homologous DNA repair of ZFN-induced breaks can render the eGFP gene in-frame with mRFP, resulting in the expression of a mRFP-eGFP fusion protein. eGFP expression was quantified using flow cytometry. (**B**) A schematic representation of the experimental procedure: After transfection with the ZFN and reporter plasmids, 293T cells were treated with various concentrations of MG132 and subjected to flow cytometry. (**C**) Flow cytometry of the cells: MG132 increased the percentage of GFP+RFP+ cells, suggesting that MG132 enhanced ZFN function. Untransfected cells and cells transfected with reporters alone were used as analysis controls. n = 3. *P<0.05.

Recently we reported a method to enrich cells with nuclease-induced mutations by transiently transfecting episomal reporters that encode fluorescent proteins and sorting the cells by flow cytometry [25] (**Figure 4A**). The reporter consists of the mRFP gene, which is constitutively expressed, and the programmable nuclease's target sequence followed by an out-of-frame eGFP gene in tandem fashion. Once a double-strand break is introduced into the target sequence by the ZFN, the eGFP gene comes into frame with mRFP because of mutations introduced by a DNA repair mechanism. The expression level of eGFP, determined by flow cytometry, represents the relative nuclease activity of the ZFN, as previously described [25]. We used this system to evaluate the effect of MG132 on ZFN activity. We transfected plasmids encoding ZFN-224 pairs, which target the *CCR5* gene, along with a reporter plasmid containing this nuclease's target site into 293T cells. At 12 hrs post transfection, the cells were split into three 35 mm dishes. The next day, the media was replaced with fresh media containing increasing concentrations of MG132 (**Figure 4B**). After 3 days of incubation at 37°C, the cells were prepared for flow cytometric analysis. The results showed that eGFP^+^ cells/mRFP^+^ cells were significantly increased on an average of 1.8-fold in the MG132 treated cells as compared to the untreated cells (**Figure 4C**). Our result indicates that MG132 treatment may enhance ZFN activity, as assayed by the ZFN-induced mutation rate in cells.

**Figure 5:**
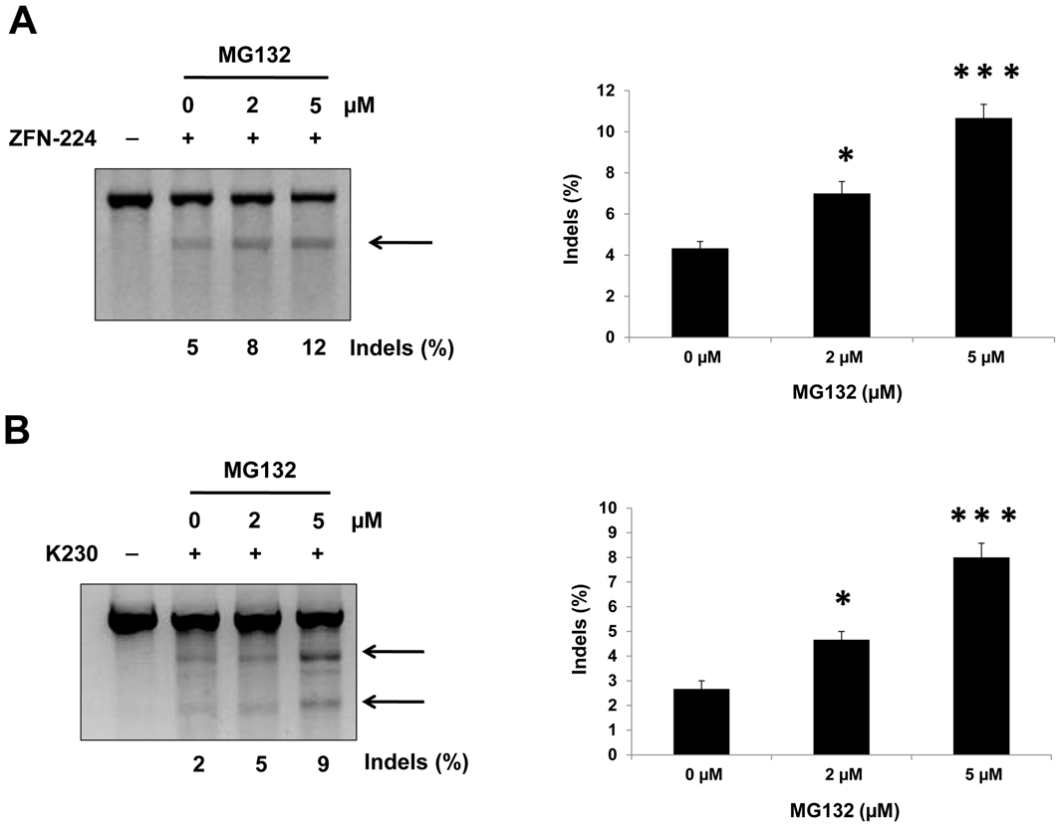
MG132 increases the frequency of ZFN-driven mutations. After transfection of plasmids encoding ZFNs (**A**: ZFN-224; **B**: K230), 293T cells were treated with various concentrations of MG132. Genomic DNA was isolated and ZFN-driven mutations were analyzed by the T7E1 assay. Arrows indicate the expected positions of DNA bands cleaved by T7E1. (**A**) ZFN-224: Because the target site lies in the center of the amplicon (780 bp), T7E1 treatment of the heteroduplexed DNA gave rise to two DNA bands with almost same size (387 bp and 389 bp), which appear as a single band. (**B**) K230: Because the target site does not lie in the center of the ampicon, T7E1 treatment of the heteroduplexed DNA gave rise to two DNA bands (493 bp and 311 bp), observed as two separate bands. The numbers at the bottom of the gel denote mutation percentages calculated by band intensities. n = 3. *P<0.05, ***P<0.001.

To validate the effect of MG132 on the frequency of ZFN-driven mutations, we isolated genomic DNA from ZFN-transfected cells that had been incubated with or without MG132 and performed a T7 endonuclease I (T7E1) assay. For experiments involving ZFN-224, we designed primers to obtain a 780 bp PCR amplicon, in which the target site lies at position 387. T7E1 treatment of the heteroduplexed DNA in the ZFN-224 group gave rise to two DNA bands of almost the same size (387 bp and 389 bp), which appear as a single band after gel electrophoresis. For experiments involving K-230, we designed primers to obtain a 806 bp PCR amplicon, in which the target site lies at position 493. T7E1 treatment of heteroduplexed DNA in the K230 group gave rise to 493 bp and 311 bp DNA fragments, which are observed as two separate bands after gel electrophoresis. The assay revealed that MG132 treatment increased the frequency of small insertions and deletions (indels) generated by K230 or ZFN-224 relative to the frequency in MG132 untreated 293T cells (**Figure 5A, B**). Similar results were also observed in HeLa cells, suggesting that the effect of MG132 is not restricted to 293T cells (data not shown). However, human embryonic stem cell lines showed showed cytotoxic response to 2 µM and 5 µM MG132 (data not shown). In the presence of MG132, the indel percentage generated by ZFNs increased by 2.5-fold (ZFN-224) or 3.0-fold (K230) when compared with that in MG132 untreated cells. Thus, the treatment of ZFN transfected cells with the proteasome inhibitor MG132 enhanced ZFN activity.

## Discussion

Targeted genetic modification using ZFNs can enable targeted gene insertion, correction, disruption, chromosomal rearrangement, and regulatory region alteration [36]. Gene editing using ZFNs is a promising technology as a powerful tool for studying biological processes and for the development of advanced gene therapy to correct pathogenic genes [17], [20], [36].

Here for the first time we investigated ZFN stability. Given that high levels of ZFN protein are associated with enhanced ZFN activity [23], [25], our protein stability study should lay the foundation for further development of ZFN technology. Furthermore, ZFNs can be now delivered directly as protein and several doses of ZFN protein treatment are required to obtain sufficient ZFN activity, because the ZFNs are degraded within a few hours after treatment [27]. Thus, the development of methods to maintain sufficient ZFN concentrations is important; our protein stability study should serve as a basis for this research. Even when a ZFN protein is continuously expressed by a DNA vector transfected into the target cells, inhibition of ZFN degradation increased the ZFN protein levels, leading to enhanced genetic modification.

Porteus' group has previously reported that short-term exposure to MG132 does not significantly increase the protein levels and activity of ZFNs that contain wild-type FokI nucleases [20], [36]. In contrast, we observed that MG132 treatment increased ZFN activity 2.4 or 2-fold. One possible reason for this discrepancy could be the different FokI nucleases employed in the two experiments: the ZFN used by Porteus' group contained the wild-type FokI nuclease, whereas we used ZFNs with a modified FokI nuclease [24], [26], which is improved from the wild type and is now predominantly used. This difference in the ZFN amino acid sequence might affect the rate of ZFN proteolysis. Another reason could be the difference in the MG132 concentration and duration of exposure: we treated cells with MG132 for 60 hours at 1, 2, and 5 µM, whereas Porteus' group used 10 µM of MG132 for only 4 hours. We observed significantly decreased cell viability at 10 µM of MG132 when cells were treated for 60 hours. In addition, the application of MG132 to human embryonic stem cells caused cytotoxic effects even at very low dosage, which is compatible with the previous reports that showed similar cytotoxic effects of MG132 on hESCs [37].

Here we showed that ZFN activity can be enhanced using a small molecule, MG132. To our knowledge, this is the first study reporting that a small molecule can regulate ZFN function. Identifying small molecules with this property is important given that ZFN technology is actively being studied as a tool for gene therapy and to analyze biological processes [36]. Although MG132 is not a FDA-approved drug, other FDA-approved proteasomal inhibitors such as bortezomib might be used together with ZFNs to enhance the effect of gene therapy. Indeed, it has been recently reported that bortezomib can increase the effect of a ZFN-expressing adeno-associated virus, although the underlying mechanism of this effect is mainly due to the enhanced transduction of the virus [20]. In addition, given that ZFN-induced gene editing is often observed only in a minor fraction of ZFN-treated cells, small molecules can be used *in vitro* to facilitate gene editing.

In conclusion, we show that ZFN proteins have a relatively short half-life and that their turn-over is regulated by the UPP. Furthermore, treatment with the proteasome inhibitor MG132 blocked ZFN protein degradation and extended its half-life, resulting in increased ZFN protein levels and enhanced genetic modification. Our protein stability study should lay the foundation for further development of ZFN technology. The identification of small molecules that increase ZFN protein levels will facilitate the application of ZFNs.

## Materials and Methods

### Cell culture and chemical reagents

293T (human embryonic kidney cell line) cells were cultured in DMEM (Gibco-BRL, Rockville, MD) supplemented with 10% fetal bovine serum (FBS, Gibco-BRL, Rockville, MD). Carbobenzoxyl-leucinyl-leucinyl-leucinal-H (MG132) (Sigma-Aldrich, St. Louis, MO), was initially dissolved in a minimal amount of DMSO to a concentration of 50 mM and aliquots were stored at −70°C. During the experiment, MG132 was thawed and diluted with DMEM culture media to a final working concentration that ranged from 0 µM to 5 µM. Cycloheximide (CHX) (Sigma-Aldrich, St. Louis, MO) was dissolved in sterile distilled water to a concentration of 10 mg/mL; a working concentration of 200 µg/mL was used in all experiments.

### Plasmids and transfections

To investigate the effect of MG132 on ZFN activity, plasmids encoding ZFNs that target chemokine receptors (CCR) genes were used in our experiments. Detailed information about ZFN-224, which targets *CCR5*, and K230, which targets a region 230 bps upstream region of *CCR5*, was previously reported [3], [34], [35]. The DNA fragment encoding ZFN-224 was sub-cloned into the pcDNA3.1/5Xmyc expression vector, a kind gift of Prof. C.H. Kim (CHA University, Seoul, South Korea). The pEFIRES-HA-ubiquitin plasmid was obtained from Prof. Yossi Yarden (Weizmann Institute, Israel). 1–2 µg of all expression constructs were transfected using polyethyleneimine (PEI) transfection reagent (Polysciences, Warrington, PA). After 6–8 hours of transfection, the media was replaced with fresh media containing the required concentration of MG132. 72 hours post-transfection, the cells were harvested and lysed using a lysis buffer (1% Triton X, 150 mM NaCl, 50 mM Tris-HCl pH 8, 1 mM PMSF) and incubated in ice for 20 mins. Western blot analysis was carried out using an anti-myc antibody (9E10, Santa Cruz Biotechnology, Santa Cruz, CA) or anti-HA antibody (Santa Cruz Biotechnology).

### Antibodies

Anti-myc (9E10) (sc-40), anti-HA (sc-7392), and anti-β-actin (sc-47778) antibodies were purchased from Santa Cruz Biotechnology.

### Treatment with reagents

293T cells, at a density of ∼2.5×10^5^ cells per well in 6 well dishes, were transfected with ZFN-encoding plasmids. After 6–8 hours of transfection, cells were treated with MG132 at a concentration ranging from 0 µM to 5 µM. At 40 hours post transfection, cells were treated with CHX at a concentration of 200 µg/mL and harvested at predetermined time intervals.

### *In vivo* ubiquitination assay

For the *in vivo* ubiquitination assay, 3 µg of pcDNA3.1-myc-ZFN-224 and pEFIRES-HA-ubiquitin were co-transfected into 293T cells. Cell lysates were immunoprecipitated with an anti-myc antibody and blotted with an anti-HA antibody. For checking the intensity of ubiquitination, co-transfected 293T cells were treated with MG132 (5 µM) for 16 hours before cell harvest. The cells were lysed in a lysis buffer (1% Triton X, 150 mM NaCl, 50 mM Tris-HCl pH 8, 1 mM PMSF) for 20 mins and centrifuged at 13,000 rpm. For immunoprecipitation, the cell lysates were incubated with appropriate antibodies (anti-myc or anti-HA) overnight at 4°C; the next day, 20 µL of protein A/G Sepharose (Santa Cruz Biotechnology, Santa Cruz, CA, USA) was added to the lysate and incubated at 4°C for 1 hour. Beads were washed with lysis buffer three times and eluted with 2X SDS loading dye. The samples were subjected to an 8% SDS-PAGE gel followed by Western blotting with anti-myc or anti-HA antibodies.

### Protein stabilization assay

293T cells were transfected with an equal amount (1 µg) of pcDNA3.1-HA-K230 48 hours after transfection, cells were treated with CHX (200 µg/mL) and periodically harvested at different time points. Equal amounts of protein from each time point were loaded onto an SDS gel for Western blot analysis.

### T7E1 assay

The T7E1 assay was performed as previously described [38]. Genomic DNA was isolated using the Genomic DNA purification Kit (Promega, Madison, WI) as per manufacturer's instructions. The region of DNA containing the programmable nuclease target site was PCR-amplified using the following primers. Z-224 (FP- 5′ GAG CCA AGC TCT CCA TCT AGT 3′; RP- 5′ CTG TAT GGA AAA TGA GAG CTG C 3′) and K230 (FP- 5′ GGG AGC TGA AAT ACC TTC CTT 3′; RP- 5′ ATG TGG CAT CAC ACA TGG AG 3′). The PCR amplicons were denatured by heating and annealed to form heteroduplex DNA, which was treated with 5 units of mismatch-sensitive T7 endonuclease 1 (New England Biolabs, Hitchin, UK) for 20 mins at 37°C and then analyzed by 2% agarose gel electrophoresis. The T7E1 treatment of heteroduplex DNA in the ZFN-224 group gave rise to 387 bp and 389 bp DNA fragments, which appear as one band after agarose gel electrophoresis, whereas that in the K230 group gave rise to 493 bp and 311 bp DNA fragments, which were observed as two separate bands after gel electrophoresis.

### Flow cytometry

Adherent cells were trypsinized and resuspended in 2% FBS in PBS. Single-cell suspensions were analyzed for RFP and eGFP signals using the FACSAria II or FACSVantage SEM (BD Biosciences, MD). Untransfected cells and cells transfected with reporters alone were used as controls [25].

### Statistical analysis

All data were expressed as means ± S.E.M. Statistical analysis was conducted using SPSS version 11.0. Student's t test was used for the statistical analysis for continuous variables between two groups and ANOVA followed by multiple comparison with Tukey's method for variables among more than two groups. A *P* value<0.05 was considered statistically significant.
